# ^68^Ga-EMP-100 PET/CT—a novel ligand for visualizing c-MET expression in metastatic renal cell carcinoma—first in-human biodistribution and imaging results

**DOI:** 10.1007/s00259-021-05596-6

**Published:** 2021-10-28

**Authors:** Lena M. Mittlmeier, Andrei Todica, Franz-Josef Gildehaus, Marcus Unterrainer, Leonie Beyer, Matthias Brendel, Nathalie L. Albert, Stephan T. Ledderose, Franziska J. Vettermann, Melanie Schott, Severin Rodler, Julian Marcon, Harun Ilhan, Clemens C. Cyran, Christian G. Stief, Michael Staehler, Peter Bartenstein

**Affiliations:** 1 Department of Nuclear Medicine, University Hospital, LMU Munich, Marchioninistr. 15, 81377 Munich, Germany; 2 Department of Urology, University Hospital, LMU Munich, Munich, Germany; 3 Department of Radiology, University Hospital, LMU Munich, Munich, Germany; 4 Institute of Pathology, LMU Munich, Munich, Germany

**Keywords:** Metastatic renal cell carcinoma, Receptor tyrosin kinase, Receptor tyrosin kinase inhibitors, c-MET, PET/CT imaging

## Abstract

**Background:**

^68^Ga-EMP-100 is a novel positron emission tomography (PET) ligand that directly targets tumoral c-MET expression. Upregulation of the receptor tyrosin kinase c-MET in renal cell carcinoma (RCC) is correlated with overall survival in metastatic disease (mRCC). Clinicopathological staging of c-MET expression could improve patient management prior to systemic therapy with for instance inhibitors targeting c-MET such as cabozantinib. We present the first in-human data of ^68^Ga-EMP-100 in mRCC patients evaluating uptake characteristics in metastases and primary RCC.

**Methods:**

Twelve patients with mRCC prior to anticipated cabozantinib therapy underwent ^68^Ga-EMP-100 PET/CT imaging. We compared the biodistribution in normal organs and tumor uptake of mRCC lesions by standard uptake value (SUV_mean_) and SUV_max_ measurements. Additionally, metastatic sites on PET were compared to contrast-enhanced computed tomography (CT) and the respective, quantitative PET parameters were assessed and then compared inter- and intra-individually.

**Results:**

Overall, 87 tumor lesions were analyzed. Of these, 68/87 (79.3%) were visually rated c-MET-positive comprising a median SUV_max_ of 4.35 and SUV_mean_ of 2.52. Comparing different tumor sites, the highest uptake intensity was found in tumor burden at the primary site (SUV_max_ 9.05 (4.86–29.16)), followed by bone metastases (SUV_max_ 5.56 (0.97–15.85)), and lymph node metastases (SUV_max_ 3.90 (2.13–6.28)) and visceral metastases (SUV_max_ 3.82 (0.11–16.18)). The occurrence of visually PET-negative lesions (20.7%) was distributed heterogeneously on an intra- and inter-individual level; the largest proportion of PET-negative metastatic lesions were lung and liver metastases. The highest physiological ^68^Ga-EMP-100 accumulation besides the urinary bladder content was seen in the kidneys, followed by moderate uptake in the liver and the spleen, whereas significantly lower uptake intensity was observed in the pancreas and the intestines.

**Conclusion:**

Targeting c-MET expression, ^68^Ga-EMP-100 shows distinctly elevated uptake in mRCC patients with partially high inter- and intra-individual differences comprising both c-MET-positive and c-MET-negative lesions. Our first clinical results warrant further systemic studies investigating the clinical use of ^68^Ga-EMP-100 as a biomarker in mRCC patients.

## Introduction

Renal cell carcinoma (RCC) is the most common kidney cancer with more than 330,000 cases diagnosed and more than 140,000 deaths worldwide every year [1]. Up to one-third of RCC patients present with synchronous metastatic spread at initial diagnosis and another third will experience tumor relapse in the further course [1–5].

The receptor tyrosine kinase c-MET regulates cell growth and differentiation as well as basic tumor pathobiology like tumor development, tumor angiogenesis, and tumor dissemination for a range of malignancies such as renal cell carcinoma [6–8]. Gibney et al. could show that higher c-MET expression was detected in all the examined samples of different RCC subtypes compared to adjacent normal renal tissue [8]. Macher-Goeppinger et al. examined c-MET expression in 572 clear cell RCC, while only 17% were negative for c-MET expression [9]. Many studies could show that upregulation of phosphorylated c-MET is correlated with larger tumor diameter, greater proliferation index, and worse overall survival [8, 10, 11].

Cabozantinib is an oral small-molecule inhibitor of tyrosine kinases (TKI) including c-MET that leads to a significantly longer progression-free and overall survival in patients with metastatic RCC (mRCC) that have progressed after VEGFR-targeted therapies [3, 12, 13]. Pretherapeutic prediction of treatment response to therapy with TKIs like cabozantinib in mRCC patients is highly desirable for the individualization of patient management and improvement of therapeutic outcomes in mRCC patients.

Unlike in most other malignancies, the application of ^18^F-fluoro-2-deoxy-2-d-glucose (^18^F-FDG) for positron emission tomography (PET) and hybrid PET imaging is of limited diagnostic yield due to low ^18^F-FDG-avidity of RCC lesions. Therefore, ^18^F-FDG-PET is not recommended by practice guidelines for RCC imaging [14, 15]. Prostate-specific membrane antigen (PSMA) and somatostatin receptor (SSTR)–targeted PET imaging are recognized as diagnostic tools in mRCC patients beyond their classical use in prostate cancer and neuroendocrine tumors, respectively [16–21].

^68^Ga-EMP-100 is a novel PET ligand based on a c-Met binding peptide (cMBP) already used in EMI-137, a clinical stage optical imaging agent. The affinity and specificity of the c-Met peptide as well as the conjugates was verified for human c-Met by fluorescence polarization (human c-Met: cMBP *K*_d_ 6.9 ± 0.8 nM, EMI-137 *K*_d_ 1.1 ± 0.8 nM, EMP-100 *K*_d_ 3.0 ± 0.5 nM, mouse *K*_d_ EMI-137: no detectable binding, dog *K*_d_ EMI-137 > 300 nM; yet unpublished data).

In addition, the cMBP was shown not competing with the native ligand and does not have a pharmacological effect on the HGF/c-Met signaling pathway either. The absence of pharmacological effect was confirmed by the absence of inhibition/activation of the receptor by the cMBP in HGF-induced proliferation assays; the absence of phosphorylation of c-Met tyrosine was observed at positions required for receptor activation, the absence of effect on tyrosine kinase activity and the phosphorylation of c-Met by HGF. Furthermore, the specificity of the peptide was demonstrated by the absence of significant affinity on a range of selective, central, and peripheral therapeutically relevant targets (70 receptors tested, yet unpublished data). The fluorescent analogue of ^68^Ga-EMP-100, EMI-137, has already been translated to clinic and comes with thoroughly documented pharmacokinetics [22, 23]. Recently, De Vries et al. could demonstrate in vivo as well as ex vivo EMI-137 as correspondent optical imaging agent to ^68^Ga-EMP-100 which targets c-MET expression in penile squamous cell carcinoma patients [24].

Hence, we hypothesized that a radioligand which directly targets c-MET could be used for the pretherapeutic estimation of c-MET expression prior to TKI therapies in mRCC, allowing for the assessment of the intra- and inter-individual heterogeneity of c-MET expression: due to the high c-MET expression in mRCC, the lack of suitability of ^18^F-FDG-PET/CT for imaging in mRCC, and the clinical need to predict therapy success in patients on therapy with cabozantinib; in the sense of therapy individualization, mRCC seems particularly suitable for c-MET imaging.

Here, we present the first in-human data of ^68^Ga-EMP-100 PET targeting c-MET peptide in mRCC patients to assess the biodistribution and intra- and inter-individual ^68^Ga-EMP-100 uptake in metastatic lesions compared to physiological uptake.

## Materials and methods

### Patients

We evaluated 12 patients with mRCC who underwent ^68^Ga-EMP-100 PET/CT. All patients gave written informed consent to undergo ^68^Ga-EMP-100 PET/CT according to the regulations of the German Pharmaceuticals Act §13(2b) and were referred for imaging by their treating urologist. There was no need for the patients to be fasting. This analysis was performed in compliance with the principles of the Declaration of Helsinki and its subsequent amendments [25], and retrospective analysis of data was approved by the institutional ethics board of the LMU Munich.

### Radiopharmaceuticals/radiosynthesis

The radiolabeling of EMP100 with ^68^Ga^3+^ obtained from a ^68^Ge/^68^Ga generator system (GalliaPharm® 50 mCi, Eckert and Ziegler AG, Berlin, Germany) was done by a simple manual synthesis under LAF conditions. Briefly, 100 μL of a stock solution of DOTA-c-MET peptide (peptide content in ultrapure water: 1 μg/μL = 0.27 nmol/μL) was mixed with 100 μL of a 3 M NaOAc). To this solution, in a 3-mL conical vial, 2 mL of ^68^Ga-eluate was added (fractionated elution, with the first 1.3 mL discarded); this labeling solution has a pH between 3.7 and 4.0. Then, the vial was heated in a heating block for 15 min at 95 °C. After 5 min of cooling, the reaction solution was taken up in a mixture of 1 mL ethanol, 1 mL phosphate buffer (sodium phosphate concentrate 20 mL, Na^+^: 1 mmol/mL, PO_4_^3−^: 0.6 mmol/mL, B. Braun, Melsungen, Germany), and 5.8 mL water for injection (WFI, Berlin Chemie, Berlin, Germany) and sterile-filtered (Millex-GV Durapore, Merck Millipore, Darmstadt, Germany). If the labeling yield was too low, the labeling solution was then purified using a C18 cartridge (SepPak C18 light, Waters Corp., Eschborn, Germany). For this purpose, the reaction mixture was taken up after heating and applied to a C18 light cartridge; the vial was rinsed with 2 mL WFI and applied to the cartridge. After rinsing with 1 mL WFI, the labeled peptide was eluted with ethanol and water (1:1); after dilution with 7 mL WFI and 1 mL phosphate buffer, sterile filtration was performed. The labeling yields were between 82 and 98% (decay-corrected, *n* = 30), and the radiochemical purity was at least 96% for all products as confirmed by radio high-performance liquid chromatography.

### PET/CT acquisition

A mean activity of 202 ± 69 MBq was injected intravenously. Additionally, the patients were premedicated with furosemide (20 mg) for radiation protection and to reduce urinary activity in the renal pelvicalyceal system if no medical contraindication was given [26].

PET was performed using a Biograph 64 PET/CT scanner (Siemens Healthineers, Erlangen, Germany). Approximately 60 min after tracer injection, the PET scan was initiated (2.5 min per bed position). The acquisition time was chosen based on pharmacokinetic data obtained in animal models (unpublished). For attenuation correction, a low-dose CT without contrast agent was acquired. Images were reconstructed iteratively using TrueX (three iterations, 21 subsets) with Gaussian post-reconstruction smoothing (2 mm full width at half-maximum).

All patients underwent a diagnostic, contrast-enhanced CT prior to ^68^Ga-EMP-100 PET/CT for staging purposes as part of the clinical routine within a mean time of 11 ± 9 days prior to ^68^Ga-EMP-100 PET/CT imaging.

### Image analysis

Image analysis was performed using a dedicated software package (Hermes Hybrid Viewer, Affinity 1.1.4; Hermes Medical Solutions, Stockholm, Sweden). Biodistribution and tumor uptake in patients were calculated by SUV_max_ and SUV_mean_ measurement.

#### Biodistribution

Organ uptake was evaluated by placing spherical volumes of interests (VOIs) inside the normal, not affected organ parenchyma using a 1-cm-diameter VOI for small organs (thyroid, parotid gland, myocardium, adrenal gland) and a 2-cm-diameter VOI for the muscle, liver, spleen, kidney, fat tissue, aortic lumen (descending aorta), lung, bone (femur), urinary bladder content, uterus, prostate, pancreas body, small intestine, and colon.

#### Tumor sites

In a first step, a visual analysis was performed; tumor lesions/metastatic sites on CT were rated visually as being either PET-avid or PET-negative by two experienced nuclear medicine physicians and two experienced radiologists. For PET quantification of tumor sites, VOI with a 50% isocontour threshold of the SUV_max_ was automatically generated around tumor lesions with focally increased tracer uptake whenever applicable. In case of close vicinity to areas with high physiological uptake or visually PET-negative lesions, a 1-cm or 2-cm spherical VOI was applied for quantification to exclude high physiological tracer excretion and to ensure reliable quantification of visually negative lesions.

Then, tumor-to-liver ratio (TLR), tumor-to-spleen ratio (TSR) and tumor-to blood pool ratio (as derived from the aorta descendens) (TBR) were calculated by dividing the SUV_max_ and the SUV_mean_ of all tumor lesions by the respective SUV_mean_ of the liver, the spleen, and the arterial blood pool.

To ensure a reliable PET quantification, small lung metastases with a SAD ≤ 0.5 cm were not included in the PET quantification analysis but reported as CT findings. In the presence of disseminated hepatic or pulmonary tumor burden, a maximum of five sites in both the PET and CT components was chosen.

### Statistical analysis

Data analysis was performed using Microsoft Excel (Microsoft, Redmond, WA, USA) and SPSS software v. 26 (SPSS Incl., IBM, Chicago, USA). Descriptive statistics are displayed as median (range) or mean ± standard deviation (SD). Kruskal–Wallis test for unpaired samples was used to determine differences of SUV_mean_, SUV_max_, TLR, TSR, and TBR between different tumor localizations. A post hoc analysis from Kruskal–Wallis testing was applied to assess differences between tumor sites; here, a Bonferroni correction was applied to counteract multiple testing. A two-tailed *p*-value < 0.05 was considered statistically significant.

## Results

### Patient characteristics

Two female and ten male patients with a median age of 64.8 (18.8–85.1) years presented for ^68^Ga-EMP-100 PET/CT at our department. One patient did not receive any local or systemic therapy prior to PET/CT, one patient underwent a single irradiation of an osteolytic metastasis in the right pelvis, while the other 10 patients underwent different systemic therapies and/or different kind of local therapies (see Table 1).

**Table 1:** Patient characteristics

No	Age (years)	Sex	Histological subtype	Number of tumor lesions/PET-negative lesions	Primary tumor resected	Localization of metastases	Previous therapies
1	73.1	Male	n.a	5/1	Yes	Lymph node, visceral, bone	Nephrectomy, local radiation, resection/CyberKnife therapy of lung metastases, sunitinib
2	58.6	Female	Chromophobe	8/3	Yes	Local recurrence, visceral, bone	Nephrectomy, resection of lung metastases, ipilimumab, nivolumab
3	85.1	Male	Clear cell	2/0	Yes	Local recurrence and vena cava tumor thrombus	Partial nephrectomy
4	56.2	Male	Clear cell	12/3	Yes	Lymph node, visceral	Nephrectomy, resection of lung metastases
5	69.0	Male	Clear cell	3/0	Yes	Lymph node	Nephrectomy, resection of lung metastases
6	60.7	Male	n.a	19/7	No	Local, lymph node, visceral, bone	Irradiation of a bone metastasis
7	83.8	Male	Clear cell	1/0	No	Bone with muscular infiltration	Irradiation spine, local CyberKnife therapy
8	70.2	Male	Clear cell	8/0	Yes	Lymph node, visceral, bone	Partial nephrectomy, partial lung resection, tivozanib, axitinib, pazopanib, nivolumab, cabozantinib, sunitinib
9	66.8	Male	Clear cell	3/0	No	Lymph node	SBRT therapy of mediastinal lymph node metastases
10	62.7	Male	Clear cell	2/0	No	Local with vena cava tumor thrombus	None
11	41.0	Female	Clear cell	9/2	No	Local, visceral, bone	SBRT of bone metastases, ipilimumab, nivolumab
12	18.8	Male	Papillary	15/2	No	Local, lymph node, visceral, bone	SBRT of bone metastases, ipilimumab, nivolumab, sunitinib

*n.a.*, not applicable

### Biodistribution of ^68^Ga-EMP-100

Calculation of SUV_mean_ and SUV_max_ was performed in the static images 1 h after injection of the radiopharmaceutical. The highest SUV_max_ and SUV_mean_ for ^68^Ga-EMP-100 PET were noted in the urinary bladder content, the kidneys, the liver, and the spleen. Lower SUV_max_ and SUV_mean_ were exemplarily observed in the pancreas body, in the small intestine and colon, and in the parotid gland. An extended overview can be found in Table 2.

**Table 2:** Biodistribution (SUV values are displayed as mean ± standard deviation)

Localization	SUV_max_	SUV_mean_
Urinary bladder content	39.7 ± 30.8	26.1 ± 13.5
Kidneys	14.4 ± 7.7	11.2 ± 7.3
Liver	5.7 ± 2.6	4.4 ± 2.5
Spleen	4.7 ± 2.7	3.7 ± 2.4
Uterus (*n* = 2)	4.1 ± 1.1	2.5 ± 0.8
Prostate (*n* = 10)	3.3 ± 1.8	1.9 ± 0.8
Aortic lumen (descendens)	3.2 ± 1.1	2.4 ± 0.9
Myocardium	2.1 ± 0.7	1.8 ± 0.6
Adrenal glands	2.1 ± 0.8	1.7 ± 0.6
Pancreas body	2.1 ± 0.9	1.5 ± 0.6
Thyroid glands	1.9 ± 0.7	1.6 ± 0.7
Small intestine	1.8 ± 1.1	1.2 ± 0.8
Colon	1.7 ± 0.8	1.2 ± 0.5
Parotid gland	1.3 ± 0.6	1.0 ± 0.5
Muscle	1.3 ± 0.7	0.9 ± 0.5
Fat tissue	0.8 ± 1.0	0.4 ± 0.2
Bone	0.6 ± 0.4	0.3 ± 0.2
Lung	0.5 ± 0.3	0.4 ± 0.2

### Tumor burden on CT and PET

6/12 (50.0%) patients had the primary tumor in situ; of these, 2/6 (33.3%) patients additionally had tumor extension into the inferior vena cava. Lymph node metastases were observed in 7/12 (58.3%) patients, visceral metastases in 7/12 (58.3%) patients, and bone metastases in 7/12 patients (58.3%).

Overall, 87 tumor lesions in 12 patients were included; among these, 8/87 (9.2%) were local tumors at the primary site and/or an infiltration of the tumor in the vena cava inferior, 20/87 (23.0%) were lymph node metastases, 39/87 (44.8%) were visceral metastases, and 20/87 (23.0%) were bone metastases. 69/87 (79.3%) were rated visually as c-MET-positive. Overall, there was a median SUV_max_ of 4.4 (0.1–29.2) and a median SUV_mean_ of 2.5 (0.1–19.1). Reporting relative quantitative values, there was a median TLR_max_ of 1.0 (0.1–5.5) and a median TLR_mean_ of 0.7 (0.1–3.6). In relation to the spleen and blood pool, there was a median TSR_max_ of 1.7 (0.2–14.0), a median TSR_mean_ of 0.9 (0.0–6.3), a median TBR_max_ of 2.1 (0.3–14.4), and a median TBR_mean_ of 1.2 (0.2–6.0).

### Primary renal tumor

**Fig. 1:**
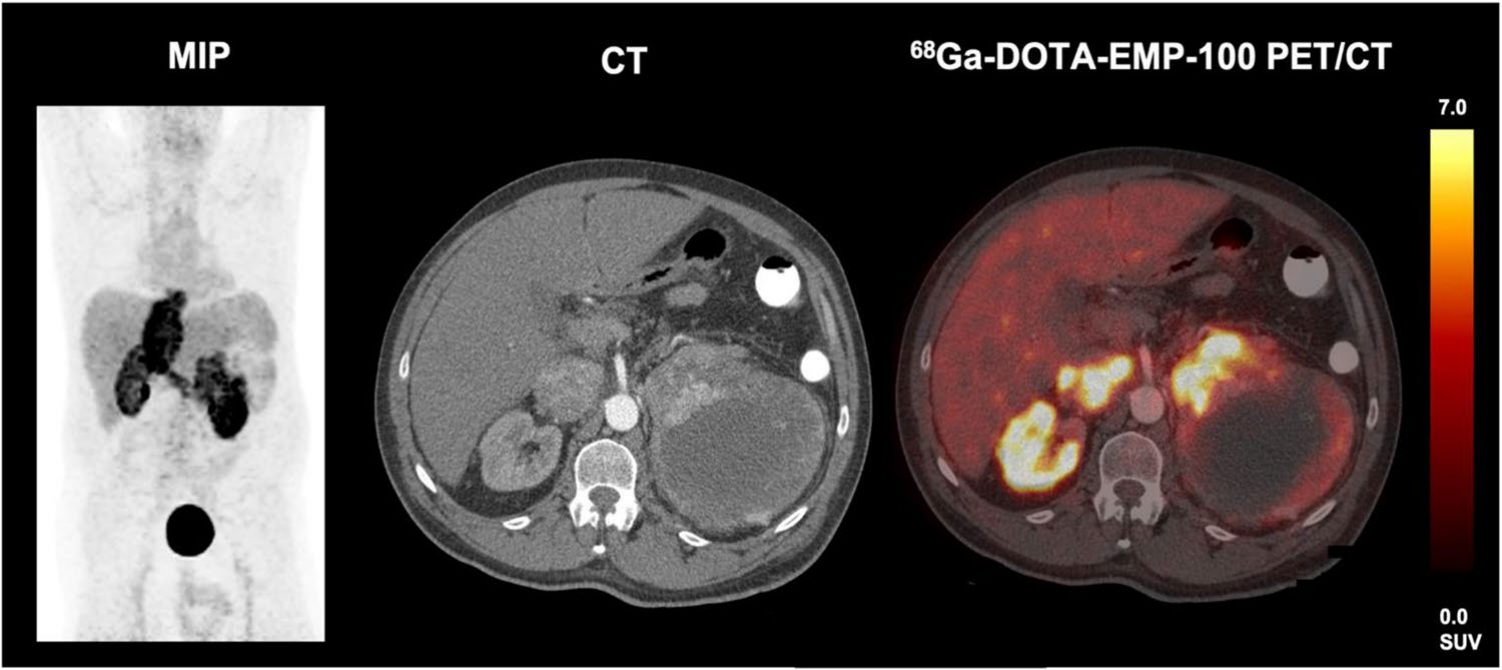
Patient with newly diagnosed mRCC and infiltration of the vena cava inferior. Heterogeneous c-MET expression of the primary tumor in the left kidney with partially highly increased and partially without any or only slightly increased ^68^Ga-EMP-100 uptake

6/12 (50.0%) patients had a primary renal tumor in situ with a median SUV_max_ of 9.1 (4.9–29.2), a SUV_mean_ of 5.8 (2.2–19.1), a TLR_max_ of 3.9 (0.4–5.5), and a TLR_mean_ of 2.2 (0.4–3.6). The median TSR_max_ was 5.0 (0.5–14.0), the TSR_mean_ was 3.3 (0.5–6.3), the median TBR_max_ was 3.8 (1.4–14.4), and the median TBR_mean_ was 2.5 (1.2–6.0). There was a heterogeneous tumoral PET-avidity in all the local tumor lesions (see also Table 3 and Fig. 1).

**Table 3:** Comparison uptake intensities of different tumor localizations (median (range))

Parameter	Local tumor burden	Lymph nodes	Visceral metastases	Bone metastases	Significance
SUV_max_	9.1 (4.9–29.2)*°	3.9 (2.1–6.3)*	3.8 (0.1–16.2)°	5.6 (1.0–15.9)	*p* = 0.001
SUV_mean_	5.8 (2.2–19.1)*°	3.2 (0.7–5.0)*	2.0 (0.1–15.1)°	2.9 (0.8–7.5)	*p* < 0.001
TLR_max_	3.9 (0.4–5.5)*°^	1.0 (0.5–2.2)°	0.9 (0.1–3.1)*	1.0 (0.1–3.0)^	*p* = 0.002
TLR_mean_	2.2 (0.4–3.6)*°^	0.7 (0.3–31.0)°	0.5 (0.1–1.5)*	0.7 (0.2–1.5)^	*p* < 0.001
TSR_max_	5.0 (0.5–14.0)*	1.0 (0.7–7.0)	1.3 (0.2–1.0)*	2.1 (0.4–5.9)	*p* = 0.005
TSR_mean_	3.3 (0.5–6.3)*°	0.7 (0.6–2.8)°	0.7 (0.1–5.0)*	1.1 (0.0–4.9)	*p* = 0.003
TBR_max_	3.8 (1.4–14.4)*	2.5 (1.1–7.2)	1.6 (0.3–10.2)*	2.1 (0.3–7.0)	*p* = 0.033
TBR_mean_	2.5 (1.2–6.0)*^	1.8 (0.6–2.8)	1.0 (0.2–5.1)*	1.1 (0.3–5.0)^	*p* = 0.002

*, °, and ^ indicate significant differences (*p* < 0.05) in the Bonferroni-corrected post hoc analysis within one quantitative parameter

### Lymph node metastases

In 7/12 (58.3%) patients, lymph nodes metastases were observed. In this group, lymph node metastases comprised a median SUV_max_ of 3.9 (2.1–6.3) and a SUV_mean_ of 3.2 (0.7–5.0), a TLR_max_ of 1.0 (0.5–2.2), and a TLR_mean_ of 0.7 (0.3–31.0). The median TSR_max_ was 1.0 (0.7–7.0), the TSR_mean_ was 0.7 (0.6–2.8), the TBR_max_ was 2.5 (1.1–7.2), and the TBR_mean_ was 1.8 (0.6–2.8) (see also Table 3). One patient (1/7, 14.3%) presented with two c-MET-negative lymph node metastases.

### Visceral metastases

In 7/12 (58.3%) patients, visceral metastases were noted. Visceral metastases showed a median SUV_max_ of 3.8 (0.1–16.2) and median SUV_mean_ of 2.0 (0.1–15.1), the median TLR_max_ was 0.9 (0.1–3.1), and the TLR_mean_ was 0.5 (0.1–1.5). The median TSR_max_ was 1.3 (0.2–10.0), the median TSR_mean_ was 0.7 (0.1–5.0), the median TBR_max_ was 1.6 (0.3–10.2), and the median TBR_mean_ was 1.0. (0.2–5.1) (see also Table 3). In these group, 5/7 (71.4%) patients showed at least one c-MET-negative metastasis in the lung and/or the liver.

### Bone metastases

7/12 (58.3%) patients comprised bone metastases. Among bone metastases, there was a median SUV_max_ of 5.6 (1.0–15.9), a median SUV_mean_ of 2.9 (0.8–7.5), TLR_max_ of 1.0 (0.1–3.0), and TLR_mean_ of 0.7 (0.2–1.5). The median TSR_max_ was 2.1 (0.4–5.9), TSR_mean_ was 1.1 (0.0–4.9), TBR_max_ was 2.1 (0.3–7.0), and TBR_mean_ 1.1 (0.3–5.0) (see also Table 3). In the group of bone lesions, in 1/7 (14.3%) patients, a single c-MET-negative bone lesion among 5 bone metastases was noted (1/5, 20.0%, not the patient that underwent single irradiation). All other bone metastases were at least slightly PET-positive.

### Correlation of different tumor localizations

The highest uptake intensity was seen in the primary tumor followed by bone metastases, lymph node metastases, and visceral metastases, e.g., median SUV_max_ 9.1 vs. 5.6 vs. 3.9 vs. 3.8, *p* = 0.001). Evaluating all quantitative parameters (SUV_max_, SUV_mean_, TLR_max_, TLR_mean_, TSR_max_, TSR_mean_, TBR_max_, TBR_mean_), the significantly highest uptake characteristics were found in tumors at the primary site. For further specifications, see also Table 3.

### Occurrence of visually PET-negative lesions

**Fig. 2:**
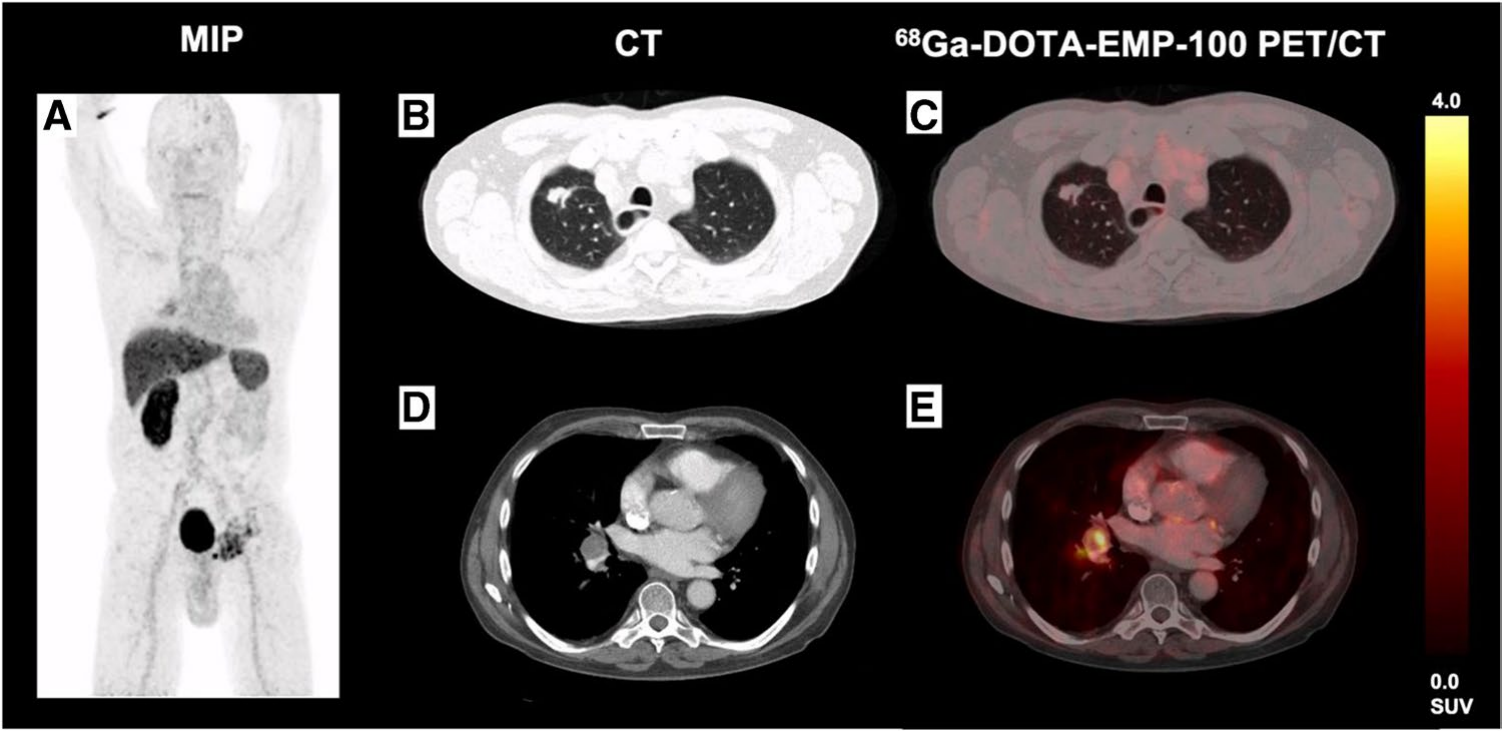
Patient with heterogeneous c-MET expression of metastatic sites in ^68^Ga-EMP-100 (see maximum intensity projection (MIP) (**A**): There was no increased uptake of ^68^Ga-EMP-100 in lung metastasis (**B**, **C**) whereas a right hilar lymph node metastasis shows moderately increased c-MET uptake (**D**, **E**)

PET-negative tumor lesions with a distinct CT-morphological correlate could be observed in 6/12 (50.0%) patients. From a total amount of 87 tumor lesions defined by CT criteria [27], 18/87 (20.7%) tumor lesions were visually c-MET-negative (sensitivity 82.9%). Of these c-MET-negative lesions, 9/18 (50.0%) were lung metastases with a SAD > 0.5 cm, 5/18 (27.8%) were liver metastases (in a patient with disseminated liver metastases), 2/18 (11.1%) were lymph node metastases, 1/18 (5.6%) was a subcutaneous soft tissue metastasis, and 1/18 (5.6%) was a bone metastasis. The occurrence of visually PET-negative lesions was very heterogeneous also within tumor localizations, i.e., there were liver metastases and lung metastases within single patients with diverging uptake characteristics with both highly PET-avid and PET-negative lesions side by side. However, there was no patient with a completely PET-negative tumor load; on the contrary, all patients comprised at least one c-MET-avid lesion (see also Fig. 2). We did not observe suspicious lesions in PET without morphological correlate on contrast-enhanced CT imaging.

## Discussion

We present the first approach for assessing c-MET expression in mRCC patients using ^68^Ga-EMP-100 PET/CT imaging. This novel ligand could provide a new promising approach to the non-invasive evaluation of c-MET expression before initiation of molecular therapies targeting c-MET such as cabozantinib or for assessing changes of c-MET expression during targeted therapies.

We evaluated the physiological biodistribution of ^68^Ga-EMP-100, as well as the tracer uptake in mRCC tumor lesions (SUV_mean_, SUV_max_) and compared the lesion uptake to the physiological uptake of liver, spleen, and blood pool. The highest physiological ^68^Ga-EMP-100 accumulation was detected inside the urinary bladder content. The high kidney uptake and the high liver uptake were most likely due to the unspecific accumulation of ^68^Ga-EMP-100. The spleen also had a moderately high ^68^Ga-EMP-100 uptake. Lower physiological uptake was seen in the small intestine, the colon, the pancreas, and the adrenal glands.

Assessing the uptake characteristics of tumor sites, our results demonstrated a high rate of PET-positive lesions with the highest uptake intensity in tumors at the primary site. Overall, the uptake intensity of ^68^Ga-EMP-100 in tumoral lesions was distinctly elevated in most of the lesions; however, given the partial renal excretion of this radioligand, a certain contribution of physiological excretion to the uptake characteristics of the tumoral masses of the primary sites has to be discussed. However, visually and quantitatively, we observed higher inter- and intra-patient uptake heterogeneity when compared to other PET-ligands such as ^18^F-PSMA-1007 or ^68^Ga-DOTATOC/^18^F-SiTATE [28, 29]. The reporting of mere SUV values seems to be a reliable parameter for the quantification of cMET-avidity of tumoral sites. In contrast, tumoral uptake compared to the physiological uptake of the liver (TLR) and the spleen (TSR) is of limited value due to the high physiological accumulation of ^68^Ga-EMP-100 in both organs. Only tumor-to-blood pool (TBR) ratios appeared to be suitable for relative quantification of tumoral sites.

We could demonstrate that the majority of tumor lesions showed a visually and quantitatively increased uptake while primary renal tumors and bone metastases showed the highest c-MET uptake. In this initial series 18/87 (20.7%), lesions did not show any visually increased uptake of ^68^Ga-EMP-100 compared to the surrounding tissue and the physiological uptake. The highest proportion of c-MET-negative lesions were liver and lung metastases: The high proportion of c-MET-negative lung metastases can at least be partly attributed to technical limitations (small size of lesions of < 1.0 cm, free breathing PET acquisition) and is consistent with other tracers [30]. On the other hand, there were numerous lung metastases over > 1.0 cm without clear c-MET uptake (see lung metastasis in Fig. 2) such that c-MET negativity per se can not only be attributed to the size of the lesions.

c-MET expression in metastatic lesions was highly heterogeneous, both on an inter- and intra-individual level; however, no patient presented completely c-MET-negative since all included patients comprised at least one c-MET-avid lesion. This variety in c-MET expression could indicate the presence of clonal heterogeneity in a single patient, but also between different mRCC patients. The intra-individual heterogeneity could also possibly be a sign of (incipient) tumor dedifferentiation with a lower c-MET uptake in some RCC cells. However, we note that immunohistochemical validation after PET imaging is needed to substantiate this claim. Nonetheless, higher c-MET uptake may result in a higher rate of response to therapy with TKI which could subsequently have an impact on the clinical outcome during therapies targeting c-MET.

**Fig. 3:**
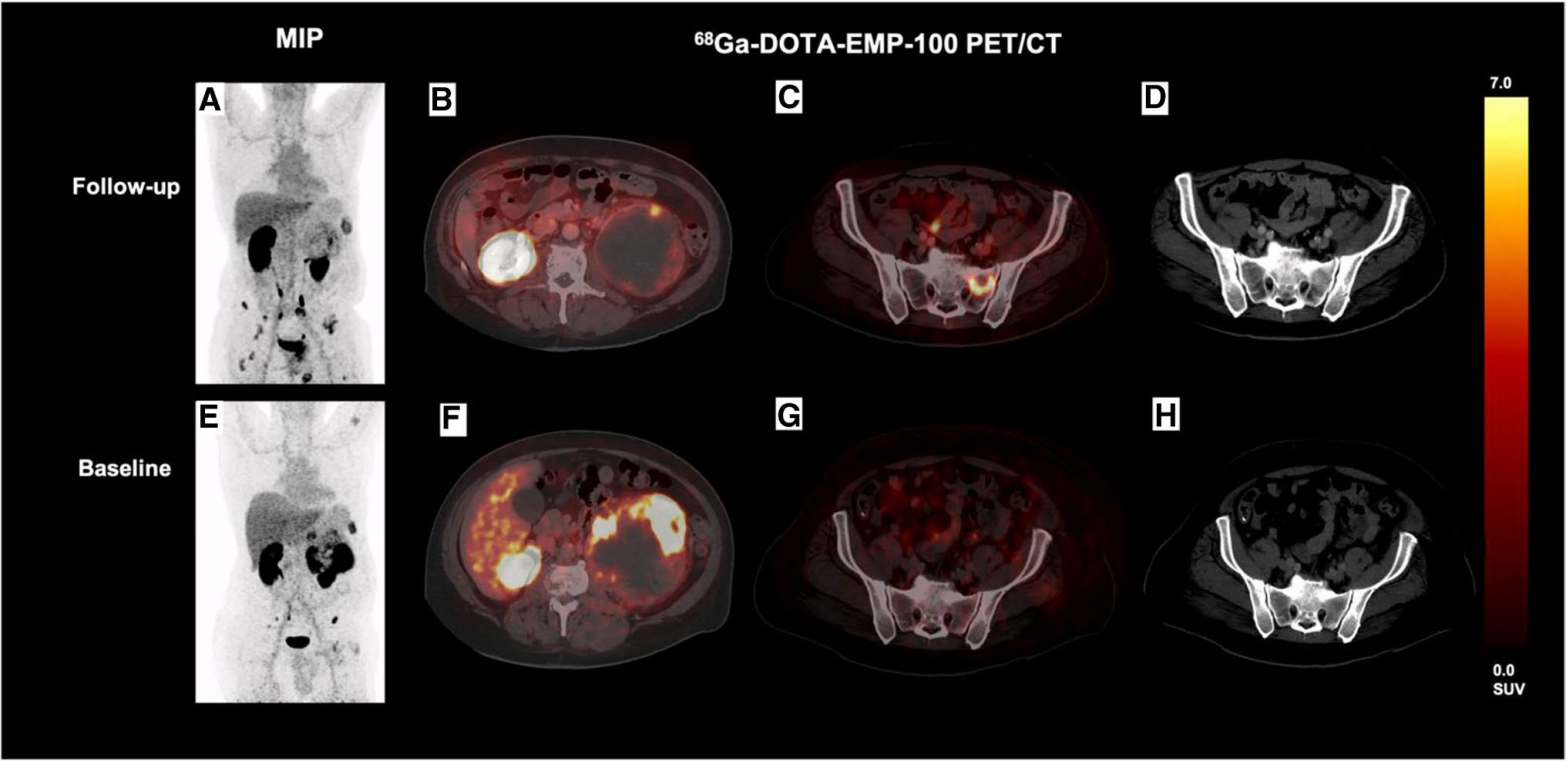
Patient with baseline (**E**–**H**) and follow-up ^68^Ga-EMP-100 (**A**–**D**). In the follow-up ^68^Ga-EMP-100, the patient showed some new lesions with low-to-moderate c-MET expression, e.g., a new c-MET-avid lesion in the left pelvis (**C**) without correlation in the CT (**D**) compared to baseline PET/CT (**G**, **H**). The primary tumor in the left kidney showed a significant size reduction with decreasing c-MET expression (**B** vs. **F**). In summary, CT assessment showed partial remission, but PET proved progressive disease in concordance with the clinical situation of the patient

Therefore, ^68^Ga-EMP-100 PET could be a promising approach for the evaluation of c-MET expression before choosing specific therapeutic agents, such as cabozantinib. Hence, PET imaging targeting c-MET expression using ^68^Ga-EMP-100 might allow a pretherapeutic estimation of treatment efficacy by non-invasive evaluation of all tumor lesions beyond the possibility of assessing the c-MET expression from a single tumor sample such as tumor resection or biopsy with the respective sampling errors in heterogeneous tumors [31]. Although current data suggests a distinct superiority of combined therapy with cabozantinib and checkpoint inhibitors [12, 32], some patients do not benefit from this approach. Therefore, PET imaging using ^68^Ga-EMP-100 might help identify patients who are most likely to respond to this combination therapy and might potentially serve as a predictive biomarker for patients with limited c-MET expression who should be treated otherwise. In the future, clinicopathological PET staging of c-MET expression using ^68^Ga-EMP-100 might also be used for monitoring changes in c-MET expression during c-MET inhibitory therapy (see Fig. 3). Thus, treatment strategies might be optimized even before clinical signs of progression, based on PET-derived signs of loss of molecular target structures. Nonetheless, it has to be noted that cabozantinib is a multitarget TKI which has multiple mechanisms of action such as VEGFR2 [33] that might have therapeutic effects beyond c-MET expression.

To sum up, ^68^Ga-EMP-100 could be used in the future to assess therapeutic options with tyrosine kinase inhibitors by evaluating the extent or even existence of c-MET expression in tumor sites before anticipated systemic treatments. Moreover, this approach might be used for response assessment during systemic therapy, whereby a therapy with tyrosine kinase inhibitors is in the foreground, obviously.

Given the labeling of EMP-100 with a DOTA-chelator, a theragnostic approach might be feasible; therefore, another potential field might be the use of cMET-PET for assessment of uptake intensity before a potential therapy with ^177^Lu-DOTA-labeled ligands targeting c-MET which is currently being investigated. Beyond the scope of mRCC, ^68^Ga-EMP-100 PET could also be used in other tumor entities with known c-MET expression such as non-small cell lung cancer or differentiated thyroid cancer [34].

A major limitation of this analysis is the retrospective design as well as the small number of patients. Further studies are needed to spatially correlate the c-MET expression with clinical follow-up as well as outcome parameters. Furthermore, in vivo and in vitro autoradiography binding studies with immunohistochemical correlation as well as preclinical studies are needed to exactly determine the c-MET expression in direct spatial correlation to the respective binding in mRCC specimens. The influence of histopathological subtypes of RCC, on the ^68^Ga-EMP-100 uptake, has yet to be established. The potential as a new tool for risk stratification based on clinicopathological characterization with PET imaging to predict therapy response needs to be evaluated in a larger trial.

## Conclusion

Visualization of c-MET expression with ^68^Ga-EMP-100 is feasible and allows for clinicopathological staging in mRCC. Our initial results warrant further studies investigating the clinical use of ^68^Ga-EMP-100 and its potential as a predictive biomarker of response to targeted therapies and outcome parameters.
